# S13-2: ”I enjoyed the camaraderie, the atmosphere and the inclusiveness of parkrun”: A qualitative study to explore mental wellbeing in middle-aged men at parkrun Ireland

**DOI:** 10.1093/eurpub/ckae114.256

**Published:** 2024-09-26

**Authors:** Allison Dunne, Helen Quirk, Alice Bullas, Steve Haake

**Affiliations:** Sheffield Hallam University, Sheffield, England; University Of Sheffield, Sheffield, England; Sheffield Hallam University, Sheffield, England; Sheffield Hallam University, Sheffield, England

## Abstract

**Purpose:**

Middle-aged men in Ireland are a population who are known to be at high risk of poor mental wellbeing and suicide. One health promotion strategy for this population is to participate in community initiatives which combine a purposeful activity with social contact to support physical, mental and social wellbeing. Research on the weekly running, walking, and volunteering initiative parkrun has indicated that participation may have mental wellbeing benefits but engagement by the population group of middle-aged men in Ireland has not been explored in depth. This study asks How does the parkrun model for community initiatives promote inclusion and mental wellbeing for middle aged men in Ireland?

**Methods:**

Semi-structured interviews were conducted online in 2022/23 with 39 men, aged 45-64 years, who run, walk or volunteer at parkrun. Men from rural and urban communities across Ireland gave interviews lasting a mean of 32 minutes. Reflexive thematic analysis was used to explore the experiences of the men during parkrun participation. Here we report the findings from one theme relating to inclusivity at parkrun; parkrun offers a supportive environment for health.

**Results:**

Many men in the study positively described the operational characteristics of parkrun such as free access, regular, weekly events, global locations, and a mix of ages and genders. Attendance with friends and family members, familiar location, a non-judgemental environment and the opportunity for socialising were all included as reasons for the welcoming atmosphere. The men described the social connections made at parkrun as being supportive for their mental wellbeing.

**Conclusions:**

The physical activity initiative parkrun can offer a supportive environment to promote mental wellbeing. As parkrun is a global initiative the benefits to other population groups at risk of poor mental wellbeing should be explored. The parkrun operational model can be applied to other physical activity initiatives to encourage inclusion and reduce barriers to participation.

**Funding:**

AD is supported by a fees-only PhD scholarship from Sheffield Hallam University

